# Transabdominal Minimally Invasive Repair of a Left Chronic Traumatic Diaphragmatic Hernia

**DOI:** 10.7759/cureus.59450

**Published:** 2024-05-01

**Authors:** Manjot Sodhi, Kevin Sigley, Kevin Jamil

**Affiliations:** 1 General Surgery, Beaumont Hospital, Dearborn, USA; 2 Bariatric Surgery, University of Rochester, New York, USA; 3 Thoracic Surgery, Corewell Health, Royal Oak, USA

**Keywords:** traumatic diaphragm injury repair, chronic diaphragm injury repair, minimally invasive repair of diaphragm, robotic repair of diaphragm, diaphragm injury

## Abstract

Although the exact incidence of traumatic diaphragmatic hernia (TDH) is unknown, it can carry significant morbidity if not treated promptly. TDH is thought to be more common in penetrating thoracoabdominal trauma compared to blunt trauma. The left side is thought to be more commonly affected than the right due to the protective effects of the liver on the right hemidiaphragm in trauma. Although large defects are evident on CT imaging and the detection rate is improved with higher resolution CT scanners, smaller ruptures may require laparoscopy for definitive diagnosis if there is a high index of suspicion. In this case report, we present a case of a missed left TDH on CT imaging, with eventual herniation of the omentum and stomach. Although TDH traditionally is approached via thoracotomy or laparotomy, we demonstrate that a transabdominal minimally invasive approach with robot-assisted laparoscopic repair is a viable option, with the potential to reduce the morbidities associated with the open approach.

## Introduction

Traumatic diaphragm rupture (TDH) is an entity of unknown incidence, reported to affect 0.8% to 6% of blunt trauma patients and more than 17% of victims of penetrating trauma to the thoracoabdominal region [1]. The left hemidiaphragm is thought to be more commonly injured due to the protective effect of the liver on the right hemidiaphragm in blunt trauma, and due to the right-handedness of the population in penetrating trauma from assault. Due to the high mechanism of injury required to rupture the diaphragm in blunt trauma, associated injuries are common, and can include solid organ laceration, rib fractures, pulmonary contusion, bowel perforation, or mesenteric disruption. Of these, rib fractures and hemopneumothorax are the most common concomitant injuries associated with TDH in blunt trauma, affecting 90% of cases, and splenic injuries are present in 27% to 60% of cases [1]. Larger defects are associated with higher injury severity scores [1], and the morbidity and mortality associated with TDH seem more related to the associated injuries rather than the diaphragmatic rupture acutely. However, in the chronic phase, TDH has the potential to cause complications associated with herniation of abdominal viscera.

TDH can be subclassified based on the timing after injury: the immediate time period between 7 and 30 days after the initial injury is termed the acute phase. The latent phase begins after recovery from the acute phase, at which time patients may develop symptoms related to the TDH. The obstructive phase begins at the time of incarceration of the hernia contents, at which time patients may develop symptoms of ischemia, obstruction, or perforation [2]. Due to the apparent high rate of progression through these phases in a missed TDH and the potential for incarceration and strangulation of abdominal viscera if not treated, TDH is recommended to be repaired upon discovery of the injury [3]. TDH is conventionally repaired via thoracotomy or laparotomy [4,5], but minimally invasive approaches have been described [6,7]. In this paper, we present a case of a chronic left TDH due to missed left TDH, with resultant herniation of the stomach.

## Case presentation

**Figure 1:**
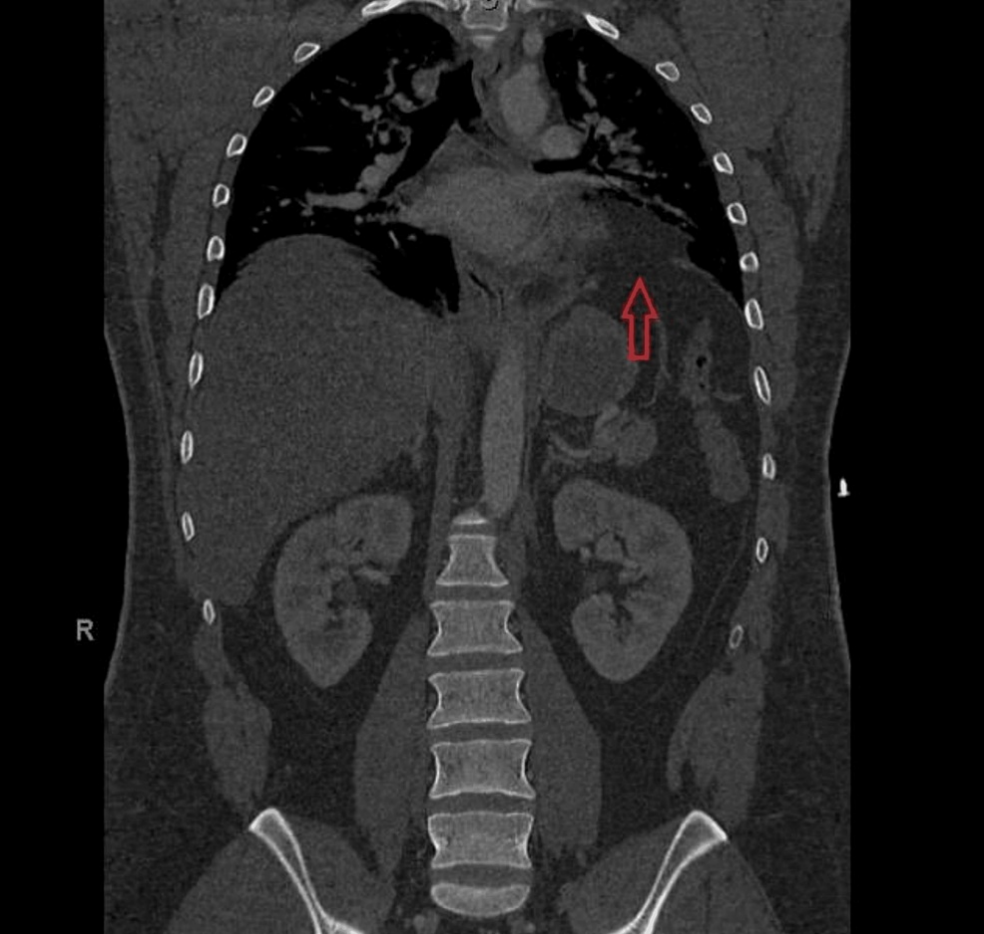
Coronal slice of CT scan on presentation, revealing left diaphragmatic discontinuity and a missed left traumatic diaphragmatic hernia CT - computed tomography

A 32-year-old male who was involved in a motor vehicle collision was brought to a level two trauma center for evaluation. He had been the driver of a car that was “T-boned” on the driver’s side. He suffered from loss of consciousness, deployment of airbags, significant intrusion into the passenger compartment, and requirement for mechanical extraction from the vehicle with the aid of a hydraulic extraction rescue tool. He reported left chest pain, left flank pain, and bilateral hip pain. On arrival in the emergency department, his Glasgow Coma Scale (GCS) was noted to be 14. The patient was noted to be slightly tachypneic and tachycardic, with vital signs of oral temperature 36.2 °C, heart rate 80 beats/min, respiratory rate 20 breaths/min, oxygen saturation (SpO_2_) 92%, and blood pressure 150/92. He was found to have tenderness of the left chest wall, left flank, and bilateral hips, as well as a 2 cm left arm laceration, and abrasions of the upper and lower extremities. He underwent computed tomography (CT) scanning, which revealed left fifth and sixth rib fractures, left pulmonary contusion, bilateral puboacetabular junction fractures, and a sacral fracture, and he was additionally diagnosed with traumatic brain injury. His chest and abdomen scans revealed a large left diaphragmatic defect (Figure 1), which was missed by the radiology and trauma teams. The patient was admitted to the hospital and his diagnosed injuries were managed without operative intervention. He was discharged to an inpatient rehabilitation facility on day four post-injury.

**Figure 2:**
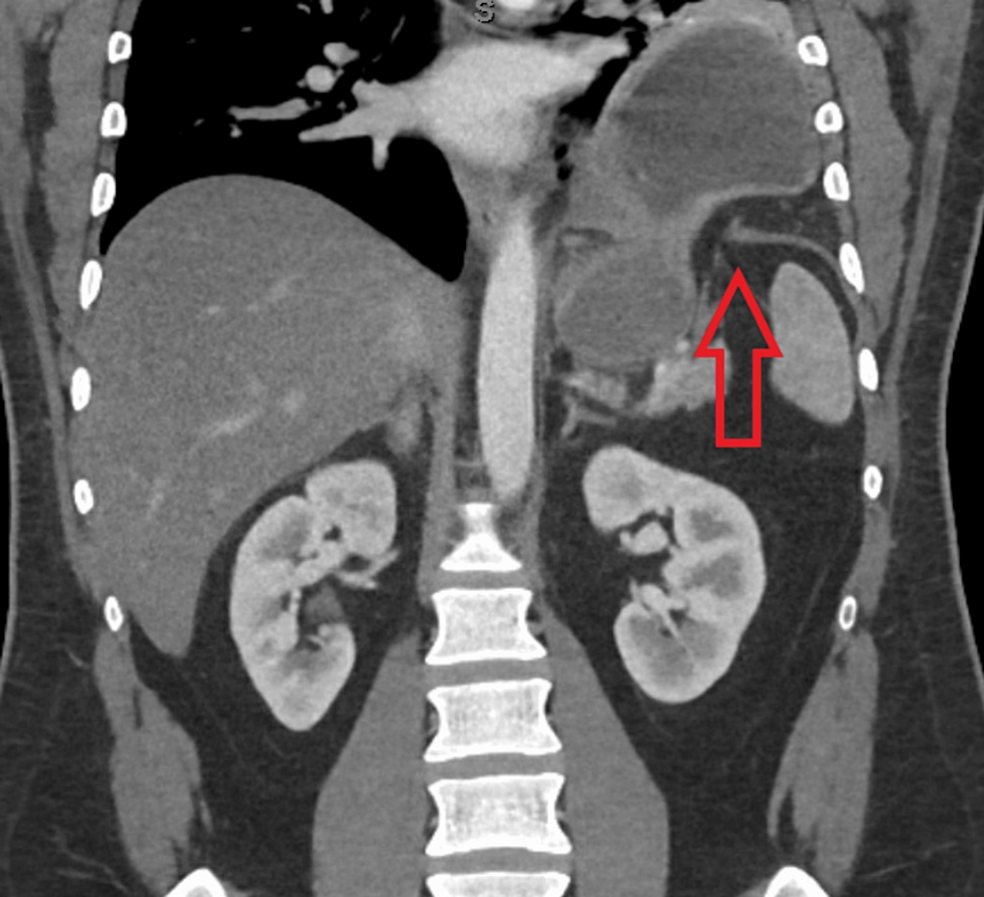
Coronal slice of CT scan two months post-injury, showing left diaphragmatic hernia with partially intrathoracic stomach. CT - Computed tomography

The patient returned to the emergency department two months after injury, with worsening left upper quadrant abdominal pain, nausea, and bloating, which were exacerbated by oral intake. He underwent CT scanning of the abdomen and pelvis which revealed a persistent large left diaphragmatic defect, with development of herniation of the stomach, but without evidence of complete obstruction or volvulus (Figure 2).

The TDH appeared to be centrally located at the dome of the left hemidiaphragm. Due to the location of the TDH, the patient’s lack of prior abdominal surgeries, low potential for intra-abdominal adhesions, and the goal of avoidance of the morbidities associated with an open approach (thoracotomy or laparotomy), the decision was made to proceed with a transabdominal laparoscopic robot-assisted approach.

**Figure 3:**
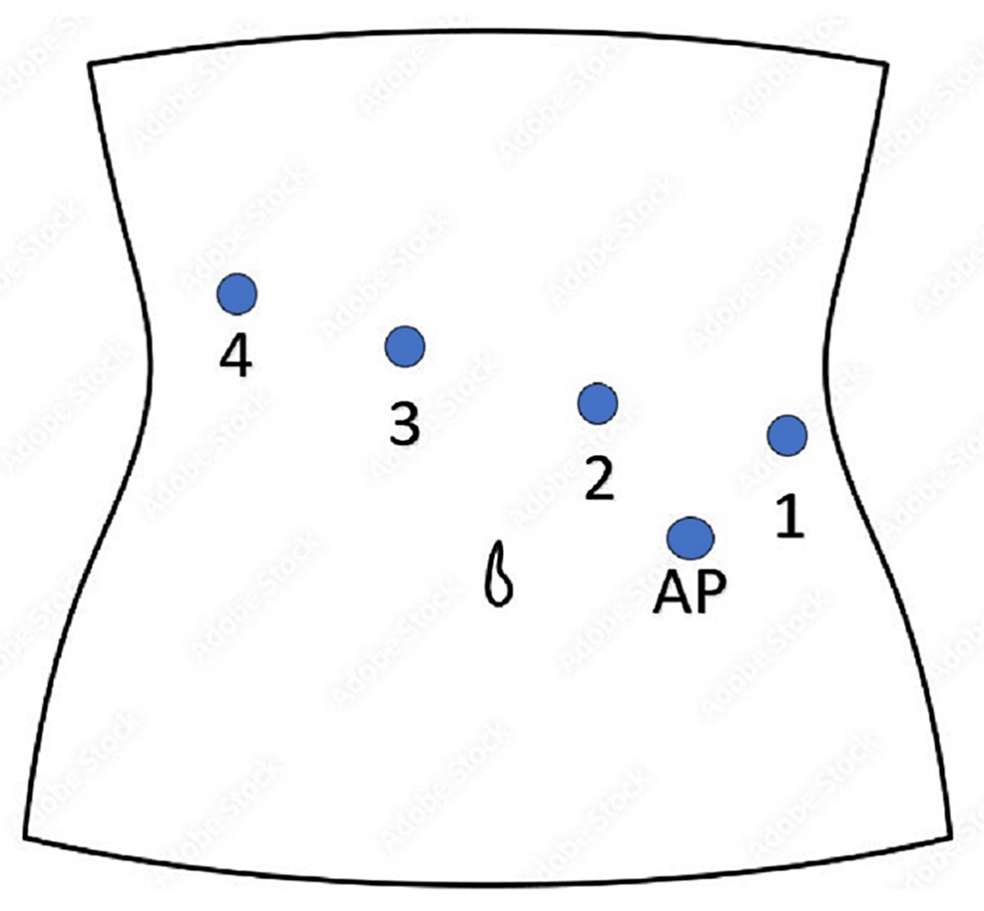
Schematic showing port placement for TDH repair 1: Arm 1, with Cadiere forceps. 2: Arm 2, with Cadiere forceps. 3: Arm 3, with 30-degree 8 mm camera. 4: Arm 4, with Maryland bipolar forceps for dissection, replaced with needle driver for mesh placement and suturing. AP: 12 mm assistant port. TDH - Traumatic diaphragmatic hernia AP - Assistant port

**Figure 4:**
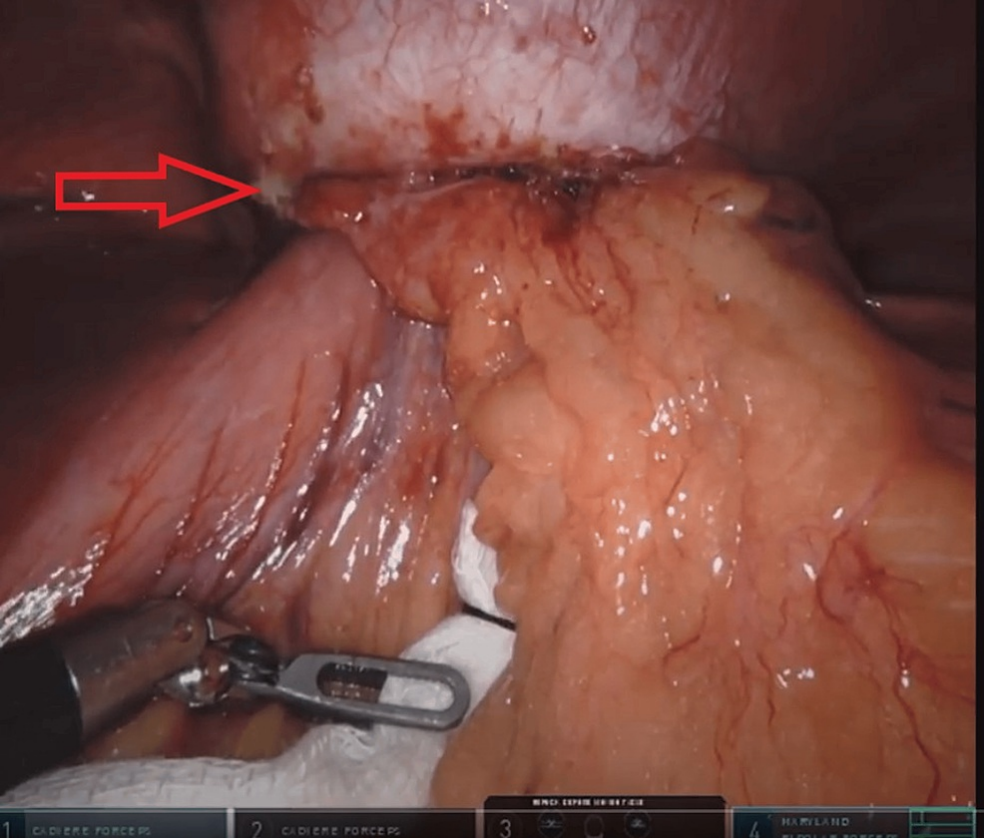
Incarcerated stomach and omentum

**Figure 5:**
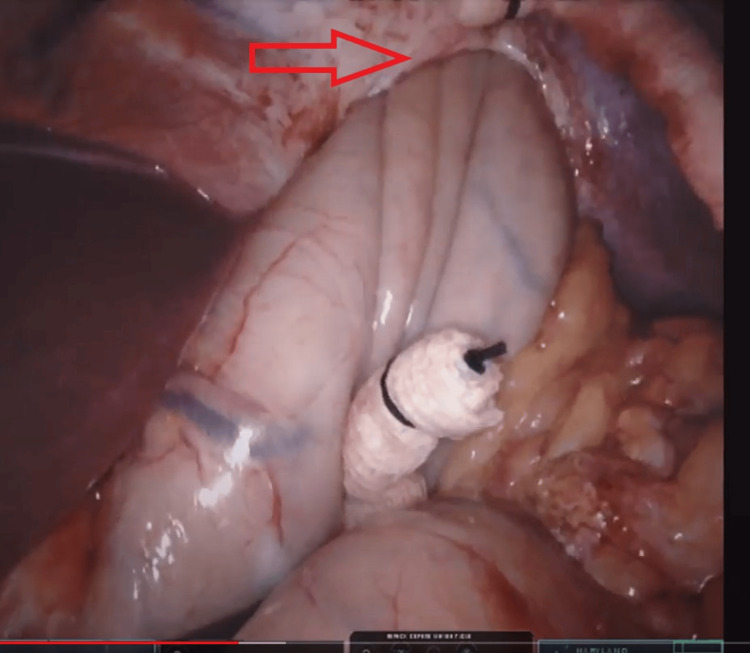
Partially reduced stomach

**Figure 6:**
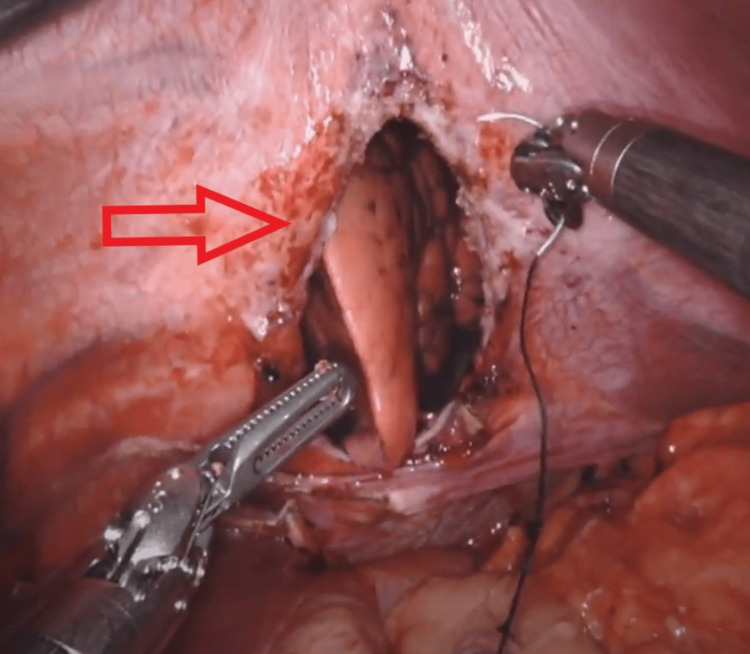
TDH after reduction of contents, before primary herniorrhaphy TDH - Traumatic diaphragmatic hernia

**Figure 7:**
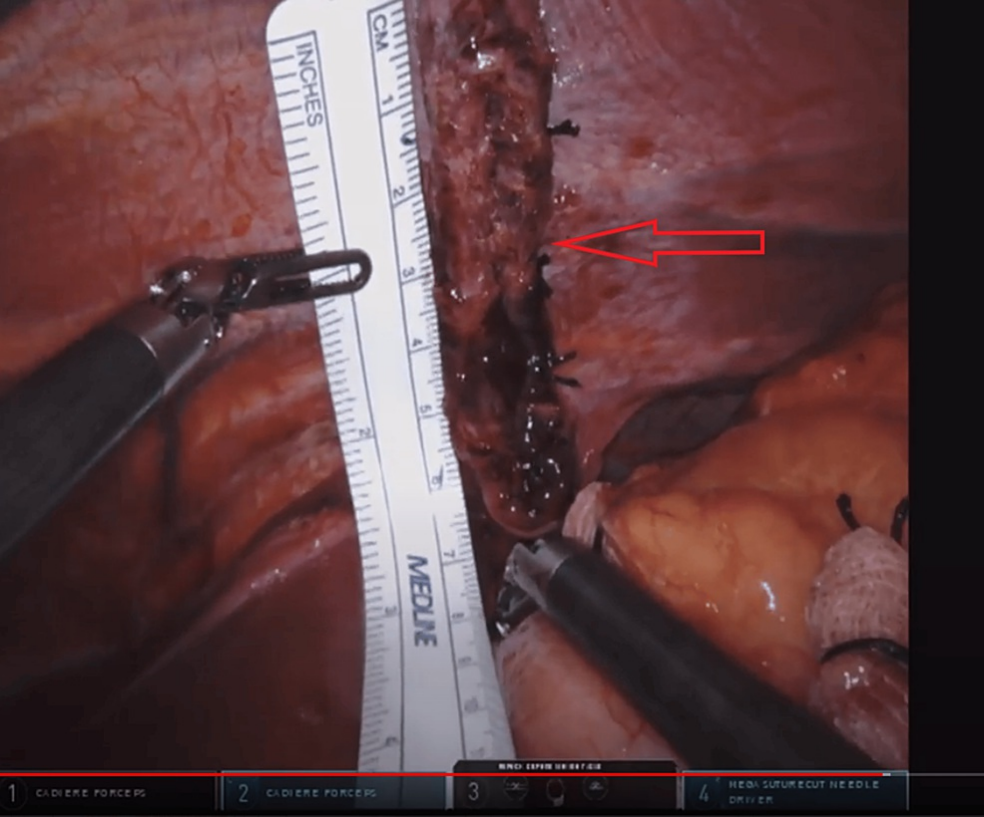
TDH repair in anterior-posterior orientation, closed with interrupted horizontal mattress ties of 0 silk suture TDH - Traumatic diaphragmatic hernia

**Figure 8:**
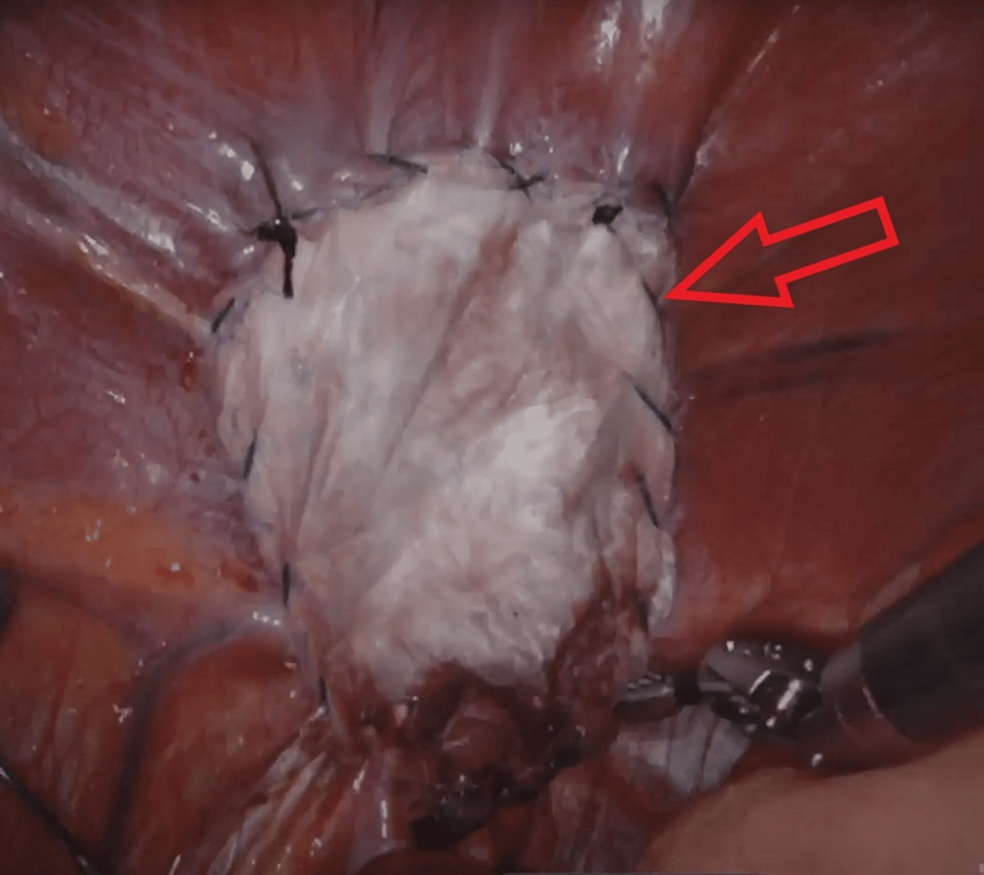
Reinforced tissue matrix mesh in place, secured with running 0 unidirectional barbed suture

Four 8 mm robotic trocars were placed in orientation about the left diaphragm and an additional 12 mm assistant port was placed inferiorly (Figure 3). The patient was placed in the reverse Trendelenburg position and the robot system was docked. Laparoscopy revealed a large defect in the center of the left diaphragm as suggested by CT imaging, containing the stomach and omentum (Figure 4). Gentle caudad traction with Caudiere forceps and rolled gauze was applied to the stomach and omentum. A bipolar Maryland forceps was used to lyse adhesions between the stomach, omentum, and diaphragm. The stomach and omentum were eventually completely reduced from the chest (Figure 5), revealing a 7 cm diameter defect, with a moderate amount of left pleural effusion, without intrathoracic adhesions. The reduced stomach and omentum appeared viable and nonischemic. The hernia defect was closed primarily using four ties of 0-silk in horizontal mattress fashion (Figure 6), in anterior-to-posterior orientation, which resulted in excellent diaphragm reapproximation (Figure 7). The decision was made to place a mesh for reinforcement with the goal of preventing hernia recurrence. A 10 cm x 12 cm reinforced tissue matrix mesh was selected, trimmed to fit, and passed through the 12 mm trocar. Two ties of 0-silk were used to anchor the mesh anteriorly and then an 0 unidirectional barbed suture was used to secure the mesh circumferentially in a running fashion. This resulted in excellent coverage and overlap of the defect (Figure 8). The fascia of the 12 mm port site was closed using a single tie of #1 braided absorbable suture. Next, to evacuate the fluid in the left chest and aid in left lung re-expansion, a left-sided 24 Fr chest tube was placed and attached to negative 20 cm H_2_O suction.

Postoperatively, the patient did very well, with a resolution of his presenting symptoms. His diet was gradually advanced, the chest tube was water-sealed and then removed, and he was discharged home on postoperative day 5.

## Discussion

TDH is a condition of unknown specific incidence. The wide range of percentages reported in both penetrating and blunt thoracoabdominal trauma likely can be attributed to the distracting nature of concomitant injuries, thin diaphragmatic muscle on cross-sectional imaging, and the potential lack of symptoms. Missed TDH is likely to lead to herniation of intraabdominal contents with the potential for strangulation and ischemic necrosis. The force required to cause a diaphragmatic rupture in blunt trauma results in nearly universal concomitant injuries, with multiple rib fractures and hemothorax or pneumothorax, the most common concomitant findings in blunt trauma victims with TDH (90%), and with splenic injury present in 27% to 60% of cases [1], whereas any penetrating injury to the thoracoabdominal region has the potential to cause left or right TDH. The rate of left TDH is conventionally thought to be higher than right TDH in both blunt and penetrating trauma. Interestingly, the examination of cadavers who are victims of trauma revealed equivalent rates of right- and left-sided TDH; however, this finding was suspected to be due to the higher levels of force resulting in death, and it does seem survivors of blunt thoracoabdominal trauma sustained TDH on the left more frequently than the right [1].

The mechanism of TDH in blunt trauma is thought to be due to the sudden increase in the pleuroperitoneal pressure gradient. The negative pressure gradient in the chest, along with a positive pressure gradient in the abdomen results in a physiologic estimated pressure differential of 7-10 cmH_2_O, which results in frequent herniation of abdominal contents if an acute TDH is not diagnosed at initial presentation [2]. A delay in diagnosis of TDH has the potential to increase mortality from 3% to 30% [7]. In cases of missed TDH, the defect is often enlarged over time due to the contraction of the muscular fibers of the diaphragm, and herniation of abdominal contents through the defect due to the pressure differential, adding to the pressure on the defect.

In the case described herein, there was clear evidence of a left diaphragmatic defect on CT imaging, but not reported on the CT interpretation, and the injury was not addressed at the initial hospital admission. The symptoms of an acute diaphragmatic rupture can include abdominal pain, chest pain, tachypnea, and vomiting. However, these symptoms are largely non-specific and can be attributed to other concomitant injuries. Additionally, patients can be entirely asymptomatic and the rupture may not be discovered until the latent or obstructive phase. Due to the large size of the defect in this case, a mesh was placed in addition to the primary repair. Mesh repair is indicated in situations where primary repair is not possible due to undue tension or other factors, and mesh may provide reinforcement to the primary repair.

Chronic TDH has traditionally been treated via laparotomy or thoracotomy. However, the laparoscopic approach (including robot-assisted, as described here) provides excellent exposure to mid-left TDH, with the potential to avoid the morbidity of an open approach [4]. A robot-assisted thoracoscopic repair has been described for a right TDH with liver herniation, with similarly excellent results [8,9], and recurrence of TDH after initial repair has also been repaired with the minimally invasive approach [10]. The advantages of a minimally invasive approach for herniorrhaphy, in general, include decreased pain, decreased wound complications, shorter hospital lengths of stay, and a more rapid return to activity compared to the open approach [11,12]. Moreover, the benefit of laparoscopy allows for the evaluation of the entire abdominal cavity, which would not be feasible with thoracotomy or thoracoscopy [5]. Major risks and limitations of the minimally invasive approach include the creation of a capnothorax with carbon dioxide (CO_2_) insufflation (via the diaphragmatic defect), adhesions of the herniated contents to the lung or upper thoracic cavity not reachable via the transabdominal laparoscopic approach, posterior diaphragmatic defects obstructed by abdominal viscera preventing exposure and repair, or CO_2_ embolism [2].

## Conclusions

TDH is a complex condition with a variety of presenting symptoms and potential complications. The concomitant injury burden is high with TDH, as well as the difficulty of identifying small defects in the thin diaphragm, may account for the not uncommon occurrence of missed TDH. Chronic TDH presents a high risk for strangulation and perforation of abdominal viscera due to the pressure differential between the thoracic and abdominal cavities. The benefits of minimally invasive approaches in general apply to TDH as well, with the avoidance of the morbidity associated with an open approach.
